# Association of Peripheral Blood Biomarkers With Response to Anti-PD-1 Immunotherapy for Patients With Deficient Mismatch Repair Metastatic Colorectal Cancer: A Multicenter Cohort Study

**DOI:** 10.3389/fimmu.2022.809971

**Published:** 2022-02-03

**Authors:** Yi-Kan Cheng, Dong-Wen Chen, Ping Chen, Xiaosheng He, Pei-Si Li, Zhen-Sen Lin, Shao-Xia Chen, Shu-Biao Ye, Ping Lan

**Affiliations:** ^1^ Department of Radiation Oncology, The Sixth Affiliated Hospital, Sun Yat-sen University, Guangzhou, China; ^2^ Guangdong Institute of Gastroenterology, Guangzhou, China; ^3^ Guangdong Provincial Key laboratory of Colorectal and Pelvic Floor Diseases, The Sixth Affiliated Hospital of Sun Yat-sen University, Guangzhou, China; ^4^ Department of Colorectal Surgery, The Sixth Affiliated Hospital, Sun Yat-sen University, Guangzhou, China; ^5^ State Key Laboratory of Oncology in South China, Guangzhou, China; ^6^ Collaborative Innovation Center for Cancer Medicine, Sun Yat-sen University Cancer Center, Guangzhou, China; ^7^ Department of VIP Region, Sun Yat-sen University Cancer Center, Guangzhou, China; ^8^ Department of Anesthesiology, Cancer Center, Sun Yat-Sen University, Guangzhou, China

**Keywords:** ratio of CD4+/CD8+, frequency of CD4+ T cell, deficient mismatch repair (dMMR), colorectal cancer (CRC), anti-PD-1 immunotherapy

## Abstract

**Purpose:**

Deficient mismatch repair (dMMR) is an established biomarker for the response to the programmed cell death (PD)-1 inhibitors in metastatic colorectal cancer (mCRC). Although patients with dMMR mCRC could achieve a high incidence of disease control and favorable progression-free survival (PFS), reported response rates to PD-1 inhibitors are variable from 28% to 52%. We aimed to explore the additional predictive biomarkers associated with response to anti-PD-1 immunotherapy in patients with dMMR mCRC.

**Methods:**

This multicenter cohort study enrolled patients with dMMR mCRC receiving anti-PD-1 immunotherapy at the Sixth Affiliated Hospital of Sun Yat-sen University and Sun Yat-sen University Cancer Center between December 2016 and December 2019. The total information of 20 peripheral blood biomarkers, including T cells (frequency of CD4+ T cell, frequency of CD8+ T cell, and ratio of CD4+/CD8+), carcinoembryonic antigen (CEA), inflammatory markers, and lipid metabolism markers, was collected. The association between response or survival and peripheral blood parameters was analyzed.

**Results:**

Among the tested parameters, the ratio of CD4+/CD8+ and frequency of CD4+ T cell were significantly associated with PFS (p = 0.023, p = 0.012) and overall survival (OS; p = 0.027, p = 0.019) in a univariate analysis. A lower level of CD4+/CD8+ ratio or frequency of CD4+ T cell showed a significant association with better overall response rates (ORRs; p = 0.03, p = 0.01). The ratio of CD4+/CD8+ and frequency of CD4+ T cell maintained significance in multivariate Cox model for PFS (HR = 9.23, p = 0.004; HR = 4.83, p = 0.02) and OS (HR = 15.22, p = 0.009; HR = 16.21, p = 0.025).

**Conclusion:**

This study indicated that the ratio of CD4+/CD8+ and the frequency of CD4+ T cell might be crucial independent biomarkers within dMMR mCRC to better identify patients for anti-PD-1 immunotherapy. If validated in prospective clinical trials, the ratio of CD4+/CD8+ and the frequency of CD4+ T cell might aid in guiding the treatment of PD-1 inhibitors among patients with dMMR mCRC.

## Introduction

Colorectal cancer (CRC) is the fourth most common cause of cancer-related death globally, and there is an increasing incidence of CRC (1, 2). DNA deficient mismatch repair (dMMR)/microsatellite instability-high (MSI-H) is a well-established biomarker for the response to programmed cell death (PD)-1 inhibitors in metastatic CRC (mCRC), for which the US Food and Drug Administration (FDA) has approved PD-1 inhibitors for treating the patients with dMMR mCRC (3). Although promising efficacy of anti-PD-1 immunotherapy has been reported in locally advanced colon cancer with dMMR tumors (4), the overall response (OR) rates (ORRs) in MSI-H mCRC patients are variable from 28% to 52% (3, 5, 6), which were likely attributed to tumor heterogeneity. Moreover, the analysis of tumor mutational burden (TMB) in tumor sampling helps to further identify MSI-H mCRC patients who respond to PD-1 inhibitors (7), but this invasive way to obtain tissues might cause treatment delay. Hence, identification of new biomarkers from the easily accessible peripheral blood is critical for selecting patients who respond better to PD-1 inhibitors.

Tumor-infiltrating lymphocytes (frequency of CD8+ T cells) mainly contribute to the antitumor immune response and are a reliable prognostic indicator for CRC (8, 9). However, it is not an optimal predictor for anti-PD-1 immunotherapy. Several peripheral blood indexes, including T cells (CD4^+^ and CD8^+^ T lymphocytes) and systemic inflammation (neutrophil-to-lymphocyte ratio (NLR), absolute neutrophil count (ANC), C-reactive protein (CRP), and lactate dehydrogenase (LDH)), have been associated with response or survival outcomes in patients with melanoma and non-small cell lung cancer (NSCLC) receiving immune checkpoint inhibitors (ICIs) (10–15). In addition, lipid metabolism has been demonstrated to play an important role in the promotion of migration (16) and invasion (17) and be related to tumor immune milieu (18). However, it remains unclear whether the peripheral blood profiling could detect the responses to anti-PD-1 immunotherapy in MSI-H mCRC patients. Thus, this multicenter study analyzed 41 mCRC patients with dMMR tumors to investigate the potential association between peripheral biomarkers with response to anti-PD-1 immunotherapy.

## Materials and Methods

### Patients

**Figure 1:**
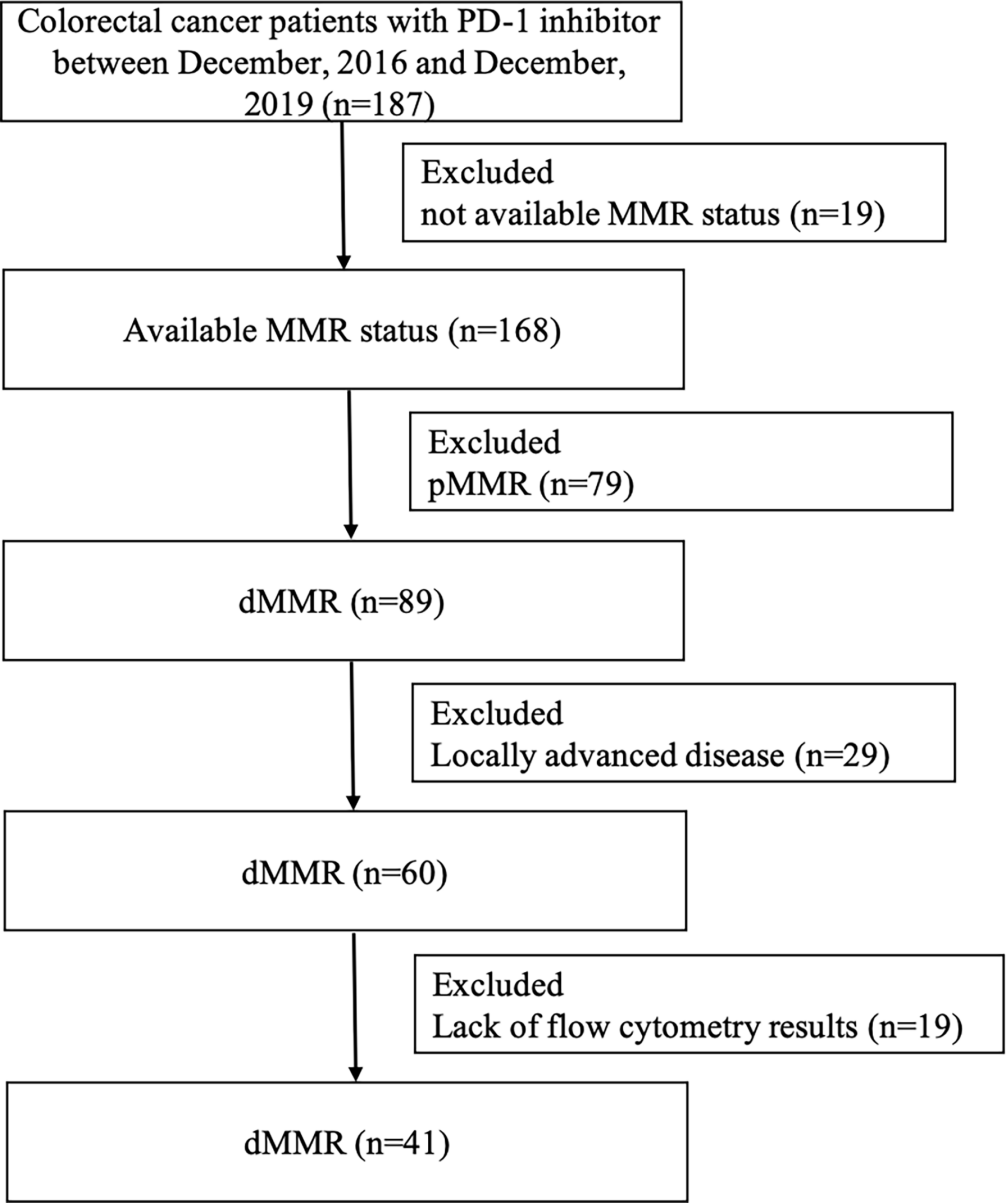
Flowchart depicting patient selection. PD-1, programmed cell death 1; MMR, mismatch repair; dMMR, deficient mismatch repair; pMMR, proficient mismatch repair.

A total of 41 mCRC patients with dMMR tumors who have been treated with anti-PD-1 inhibitor (nivolumab, pembrolizumab, triprizumab, toripalimab, and camrelizumab) were identified at the Sixth Affiliated Hospital of Sun Yat-sen University and Sun Yat-sen University Cancer Center between December 2016 and December 2019 (**Figure 1**). The end of the follow-up was June 30, 2020. The study was approved by the institutional review board of the Sixth Affiliated Hospital of Sun Yat-sen University. Written informed consent from patients was waived due to the retrospective nature of our study (**Table 1**).

**Table 1:** Patients’ characteristics.

Characteristics	No. (%) of Patients	CR/PR	SD/PD	p-Value^a^
	(n = 41)	(n = 23)	(n = 18)	
Age, years, median (range)	41 (20–77)	35 (20–68)	47 (21–77)	0.16
Gender				0.13
Male	22 (54)	10 (43)	12 (67)	
Female	19 (46)	13 (57)	6 (33)	
Grade				0.40
High	5 (12)	4 (17)	1 (6)	
Moderate	14 (34)	8 (35)	6 (33)	
Low	15 (37)	6 (26)	9 (50)	
NA	7 (17)	5 (22)	2 (11)	
Tumor location				0.73
Colon	30 (73)	16 (70)	14 (78)	
Rectum	11 (27)	7 (30)	4 (22)	
Known KRAS status^b^				1.0
Mutant	16 (73)	8 (73)	8 (73)	
Wild-type	6 (27)	3 (27)	3 (27)	
Known BRAF status^c^				1.0
Mutant	2 (9)	1 (9)	1 (9)	
Wild-type	20 (91)	10 (91)	10 (91)	
Frequency of CD4+ T cells, %, median (range)	37 (23–61)	32 (23–51)	41 (25–61)	0.013
Frequency of CD4+ T cells, %				0.01
>39.5	16 (39)	5 (22)	11 (61)	
≤39.5	25 (61)	18 (78)	7 (39)	
Frequency of CD8+ T cells, %, median (range)	27 (12–53)	28 (15–53)	24 (12–46)	0.24
Ratio of CD4/CD8, %, median (range)	1.3 (0.5–4.6)	1.1 (0.5–2.3)	1.9 (0.6–4.6)	0.12
Ratio of CD4/CD8, %				0.03
>1.64	15 (37)	5 (22)	10 (56)	
≤1.64	26 (63)	18 (78)	8 (44)	
CEA, ng/ml, median (range)	9.3 (1.1–754.6)	5.0 (1.4–754.6)	44.0 (1.1–596.1)	0.03
CRP, mg/L, median (range)	14.4 (0.2–201.7)	12.5 (0.2–201.7)	16.3 (0.5–181.8)	0.47
LDH, U/L, median (range)	197.2 (130.9–931.2)	171.9 (135.5–931.2)	228.0 (130.9–567.3)	0.19
Neutrophils, 10E9/L, median (range)	4.1 (0.6–20.7)	3.3 (0.6–10.9)	4.7 (1.2–20.7)	0.17
Lymphocytes, 10E9/L, median (range)	1.3 (0.3–2.8)	1.3 (0.3–2.2)	1.3 (0.5–2.8)	0.88
NLR, median (range)	3.3 (0.6–26.0)	2.9 (0.6–26.0)	3.6 (1.0–17.6)	0.34
Monocytes, 10E9/L, median (range)	0.6 (0.2–1.8)	0.5 (0.2–1.8)	0.6 (0.3–1.0)	0.72
Platelets, 10E9/L, median (range)	272.0 (111.6–479.7)	265.0 (111.6–444.0)	276.9 (126.0–479.7)	0.82
PLR, median (range)	180.5 (61.5–900.0)	180.5 (61.5–900.0)	182.7 (66.8–622.4)	0.94
LMR, median (range)	2.1 (0.5–9.3)	2.1 (0.5–9.3)	2.2 (1.1–7.7)	0.81
ALB, g/L, median (range)	40.8 (23.0–49.5)	40.9 (26.4–49.5)	40.5 (23.0–48.1)	0.62
CHO, mmol/L, median (range)	4.4 (3.4–6.8)	4.8 (3.5–6.8)	4.0 (3.4–5.2)	0.45
TG, mmol/L, median (range)	1.2 (0.5–3.9)	1.1 (0.7–3.9)	1.4 (0.5–3.4)	0.79
HDL, mmol/L, median (range)	1.2 (0.5–5.1)	1.3 (0.6–5.1)	1.1 (0.5–1.7)	0.11
LDL, mmol/L, median (range)	2.8 (0.8–7.7)	2.8 (1.2–3.8)	2.4 (2.0–7.7)	0.63
ApoA1, g/L, median (range)	1.2 (0.3–1.7)	1.2 (0.7–1.7)	1.1 (0.3–1.5)	0.29
ApoB, g/L, median (range)	0.8 (0.4–1.5)	0.9 (0.4–1.5)	0.8 (0.4–1.2)	0.45

CR, complete response; PR, partial response; SD, stable disease; PD, progressive disease; CEA, carcinoembryonic antigen; CRP, C-reactive protein; LDH, lactate dehydrogenase; ALB, albumin; NLR, neutrophil-to-lymphocyte ratio; PLR, platelet-to-lymphocyte ratio; LMR, lymphocyte-to-monocyte ratio; CHO, cholesterol; TG, triglyceride; HDL, high-density lipoprotein; LDL, low-density lipoprotein; ApoA1, apolipoprotein A1; ApoB, apolipoprotein B.

a p-Values were estimated by Fisher’s exact test and Mann–Whitney U test for categorical variables and continuous variables, respectively.

b A total of 22 patients were tested with KRAS.

c A total of 22 patients were tested with BRAF.

Pretreatment clinicopathologic features and treatment history were collected from the individual database at these two institutions, which included age, sex, stage, tumor location, histologic subtype, carcinoembryonic antigen (CEA), mutational status (KRAS and BRAF), T cells [CD4+ T cell (CD3+ CD4+ T cell), CD8+ T cell (CD3+CD8 T cell), and ratio of CD4+/CD8+], inflammatory biomarkers [neutrophils, lymphocytes, monocytes, platelets, NLR, platelet-to-lymphocyte ratio (PLR) and lymphocyte-to-monocyte ratio (LMR), LDH, CRP, and albumin (ALB)], and lipid metabolism markers [cholesterol (CHO), triglyceride (TG), low-density lipoprotein (LDL), high-density lipoprotein (HDL), apolipoprotein A1 (ApoA1), and apolipoprotein B (ApoB)]. Pretreatment values were defined as those obtained before the initiation of anti-PD-1 immunotherapy.

### Flow Cytometry

**Figure 2:**
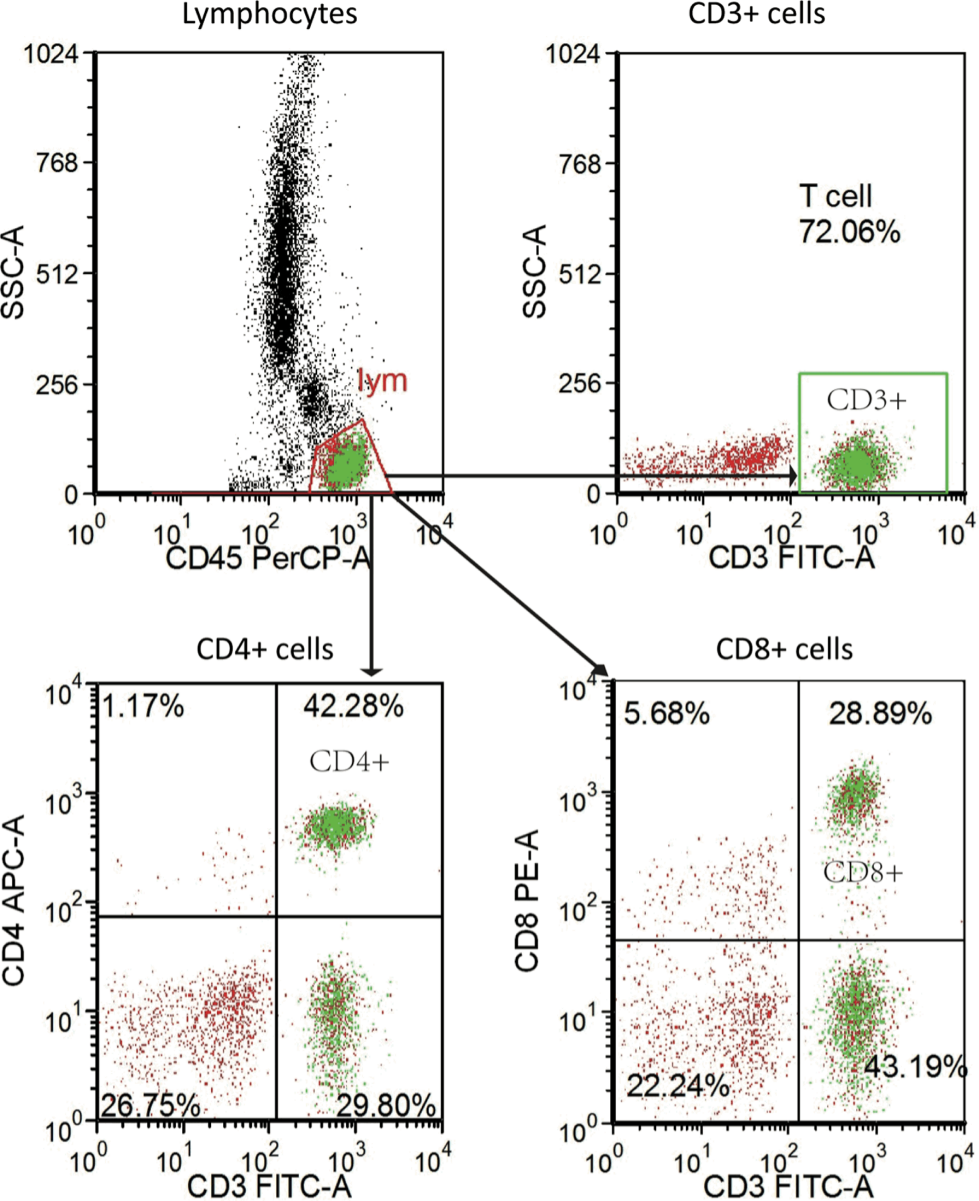
Representative flow-cytometry gating strategy for quantifying the numbers of various immune cell subsets in PBMC. PBMC, peripheral blood mononuclear cell.

We obtained the peripheral blood samples before patients received anti-PD-1 immunotherapy. The antibodies for staining were Ab anti‐CD4 (APC-labeled CD4, clone SK3), anti‐CD8 (PE-labeled CD8, clone SK1), anti‐CD3 (FITC-labeled CD3, clone SK7), and anti‐CD45 (PerCP-labeled CD45, clone 2D1 [HLe-1]). All the above Abs (BD Biosciences, San Jose, CA, USA) included isotype‐matched negative controls. Well-mixed, anticoagulated whole blood measuring 100 µl was vortexed gently with 20 µl of abs and was incubated for 15 min in the dark at room temperature according to the procedure of BD Multitest™ CD3/CD8/CD45/CD4 kit (No. 340499, BD, USA). A total of 450 µl of 1× BD FACS lysing solution was then added and incubated for 15 min in the dark at room temperature. The stained cells were analyzed on a BD FACS Canto II flow cytometry system with FACS Diva software (BD Biosciences) (**Figure 2**).

### Statistical Analysis

Tumor response was evaluated according to the Response Evaluation Criteria in Solid Tumors (RESICT), version 1.1. The disappearance of all target lesions was defined as complete response (CR). Baseline sum diameters were taken as reference according to the RESICT criteria. Partial response (PR) was defined as at least a 30% decrease in the sum of diameters of target lesions. Progressive disease (PD) was defined as at least a 20% increase in the sum of diameters of target lesions. In addition to the relative increase of 20%, the sum must also demonstrate an absolute increase of at least 5 mm. Neither sufficient shrinkage to qualify for PR nor sufficient increase to qualify for PD was defined as stable disease (SD). Progression-free survival (PFS) was defined as the duration from the date of immunotherapy initiation to clinical or radiographic progression or death. Overall survival (OS) was defined as the duration from the date of immunotherapy initiation to death. Fisher’s exact test and Mann–Whitney U test were performed to compare distribution between groups based on response for categorical variables and continuous variables, respectively. Univariate Cox regression model was performed to estimate the hazard ratios (HRs) and 95% CIs of survival based on clinicopathologic parameters and peripheral blood indexes. The receiver operating characteristic (ROC) curve analysis was used to determine the cutoff point for the continuous variables including peripheral blood parameters. The Kaplan–Meier method was used to perform survival analysis, with p-values compared by the log-rank test. Only parameters with statistical significance in a univariate analysis were included in multivariable analysis. HRs and 95% CIs of survival were estimated by multivariate Cox regression models. A two-tailed p-value <0.05 was considered statistically significant. All statistical analyses were performed in R software (version 3.5.1; http://www.Rproject.org).

## Results

### Patients Characteristics

A total of 41 mCRC patients with dMMR tumors were identified to be treated with PD-1 inhibitors. Clinical outcomes are depicted in **Table S1**. Overall, 4 patients achieved a CR, 19 patients achieved a PR, 10 patients achieved SD, and 8 patients achieved a PD, which led to an ORR of 56% (23/41). The details of the patients’ clinicopathologic characteristics are shown in **Table 1**. The median age for the entire cohort was 41 years (range 20–77), and 54% of patients were male. KRAS mutations were observed in 73% (16/22) of patients, while BRAF mutations in 9% (2/22). A total of 30 patients (73%) had colon tumors. The median values of the frequency of CD4+ T cells, frequency of CD8+ T cell, and ratio of CD4+/CD8+ for the entire cohort were 37 (23–61), 27 (12–53), and 1.3 (0.5–4.6), respectively.

### Association Between Biomarkers and Objective Response

Characteristics and OR were compared between responders (CR/PR) and non-responders (SD/PD). The frequency of CD4+ T cell and CEA as continuous variables were significantly associated with ORR (all the p-values <0.05, **Table 1**), while other investigated parameters were similar despite the significant association of a lower level for the ratio of CD4/CD8 T cells with ORR (p = 0.03). For mutation data, KRAS or BRAF mutations did not show any significant difference (**Table 1**).

### Association Between Biomarkers and Survival

**Figure 3:**
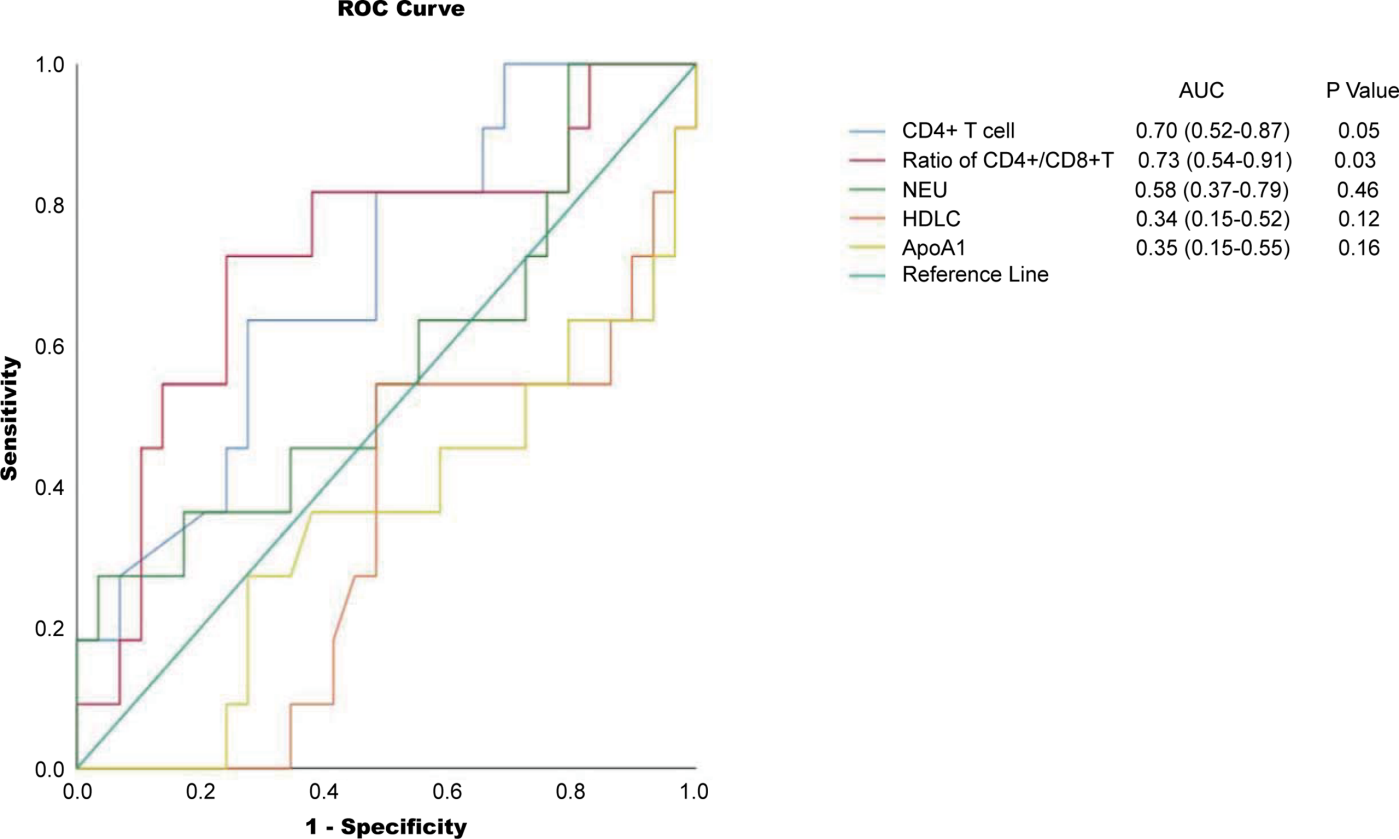
ROC curves for the cutoff points for frequency of CD4+ T cell, ratio of CD4+/CD8+, NEU, HDL, and ApoA1. NEU, neutrophils; HDL, high-density lipoprotein; ApoA1, apolipoprotein A1; ROC, receiver operating characteristic.

**Figure 4:**
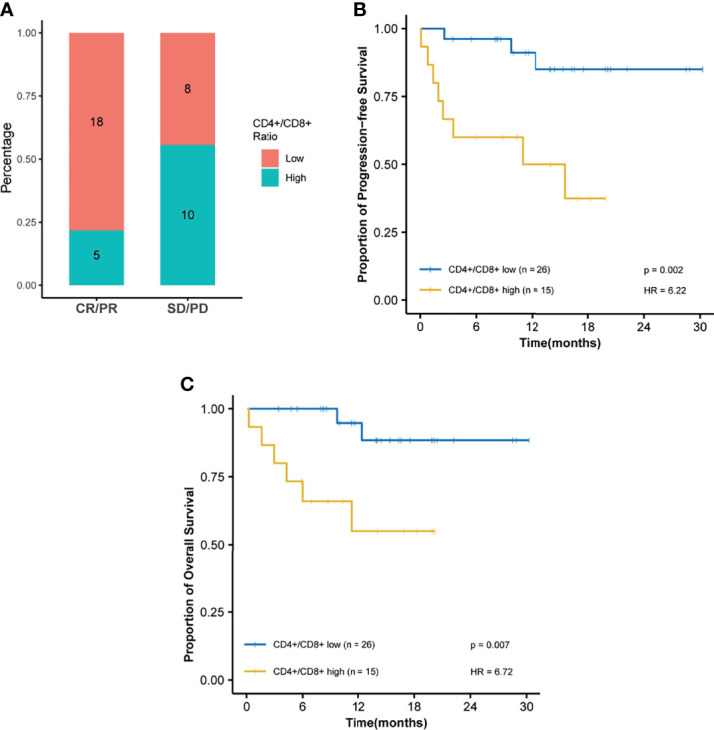
The ratio of CD4+/CD8+ is predictive of response and survival outcome. Optimal cutoff point was calculated by receiver operating characteristic (ROC) curve analysis to dichotomize patients into high and low groups. **(A)** Ratio of CD4+/CD8+ distribution is visualized by a histogram between treatment response groups (CR, complete response; PR, partial response; SD, stable disease; PD, progressive disease). **(B)** Kaplan–Meier survival curves for progression-free survival. **(C)** Kaplan–Meier survival curves for overall survival.

**Figure 5:**
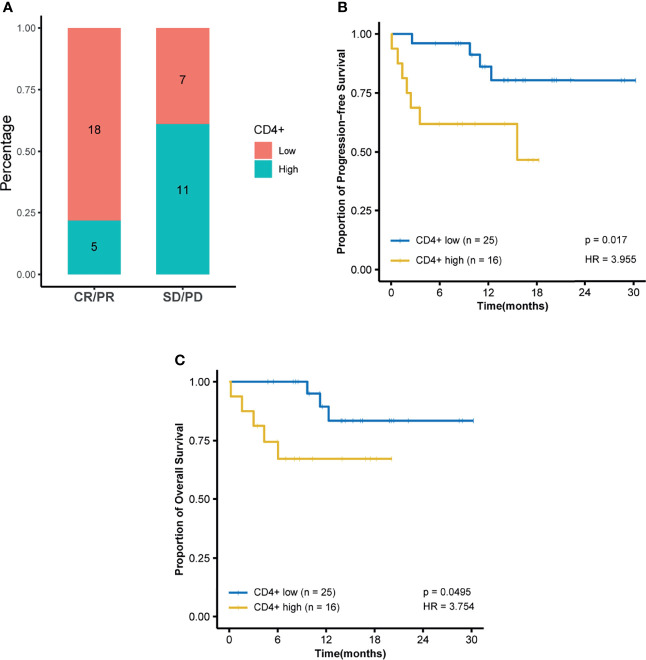
Frequency of CD4+ T cell is predictive of response and survival outcome. Optimal cutoff point was calculated by receiver operating characteristic (ROC) curve analysis to dichotomize patients into high and low groups. **(A)** Frequency of CD4+ T-cell distribution is visualized by a histogram between treatment response groups (CR, complete response; PR, partial response; SD, stable disease; PD, progressive disease). **(B)** Kaplan–Meier survival curves for progression-free survival and **(C)** Kaplan–Meier survival curves for overall survival.

Among all tested parameters correlated with PFS, gender, age, tumor location, tumor grade, stage, and KRAS as well as BRAF status did not affect PFS or OS by using a Cox regression model (**Tables 2**, **S2**). The frequency of CD4+ T cell, ratio of CD4+/CD8+, HDL, and ApoA1 were associated with PFS in a univariate Cox regression model (**Table 2**). The frequency of CD4+ T cell, ratio of CD4+/CD8+, NLR, HDL, and ApoA1 were associated with OS in a univariate Cox regression model (**Table S2**). With the use of ROC curves, the cutoff values of the above variables for PFS were identified (**Table 2** and **Figure 3**). The potential survival-related factors (HDL, ApoA1, and NLR) were not significantly associated with the frequency of CD4+ T cell or ratio of CD4+/CD8+ (**Table S3**). The frequency of CD4+ T cell and ratio of CD4+/CD8+ remained significant in a multivariate analysis for both PFS and OS (**Tables 3**, **S4**). The optimal predictive cut-points of CD4+/CD8+ ratio and frequency of CD4+ T cell were 1.64 and 39.5, respectively. For the group with a low level of CD4+/CD8+ ratio, 18 of 26 (69%) cases had an OR (CR+PR), while only 5 of 15 (33%) had an OR (p = 0.03) for the group with a higher value of CD4+/CD8+ ratio (**Table 1** and **Figure 4**). Log-rank analysis revealed that a lower level of CD4+/CD8+ ratio was associated with a better PFS (p = 0.002) and OS (p = 0.007) (**Figure 4**). In a multivariate analysis, the ratio of CD4+/CD8+ remained significant in predicting PFS (p = 0.004, HR = 9.23, 95% CI = 2.04–41.7) and OS (p = 0.009, HR = 15.22, 95% CI = 2.00–115.8) (**Tables 3**, **S3**). For the group with a lower level of the frequency of CD4+ T cell, 18 of 25 (72%) cases had an OR (CR+PR), while only 5 of 16 (31%) had an OR (p = 0.01) for the group with a higher value of the frequency of CD4+ T cell (**Table 1** and **Figure 5**). Log-rank analysis revealed that a lower level of the frequency of CD4+ T cell was associated with a better PFS (p = 0.017) and OS (p = 0.0495) (**Figure 5**). In a multivariate analysis, the frequency of CD4+ T cell remained significant in predicting PFS (p = 0.02, HR = 4.83, 95% CI = 1.28–18.27) and OS (p = 0.025, HR = 16.21, 95% CI = 1.43–184.2) (**Tables 3**, **S4**). Furthermore, NLR was significantly associated with OS in both univariate and multivariate analyses.

**Table 2:** Progression-free survival and associations with clinicopathologic features using Cox regression.

Clinicopathologic Parameters	HR	95% CI	p-Value
Age (years)			
Continuous	1.03	0.99–1.07	0.11
Gender			
Female versus male	1.74	0.51–6.0	0.38
Location			
Rectum versus colon	0.53	0.16–1.83	0.32
Grade			
High versus moderate/low	0.038	0.0–16.09	0.29
KRAS mutation			
Yes versus no	0.30	0.06–1.50	0.14
BRAF mutation			
Yes versus no	0.04	0.00–2,165.88	0.56
Frequency of CD4+ T cell^a^ (%)			
Continuous	1.09	1.02–1.16	0.012
>39.5 versus ≤39.5	4.05	1.17–13.97	0.027
Frequency of CD8+ T cell^a^ (%)			
Continuous	0.94	0.87–1.01	0.09
Ratio of CD4+/CD8+^a^ (%)			
Continuous	1.81	1.08–3.01	0.023
>1.64 versus ≤1.64	5.99	1.58–22.70	0.008
CEA (ng/ml)			
Continuous	1.002	1.00–1.004	0.09
CRP (mg/L)			
Continuous	1.007	0.997–1.02	0.15
LDH (U/L)			
Continuous	1.002	0.999–1.004	0.26
Neutrophils^a^ (10E9/L)			
Continuous	1.27	1.09–1.49	0.002
>4.35 versus ≤ 4.35	1.14	0.35–3.76	0.82
Lymphocytes (10E9/L)			
Continuous	0.77	0.25–2.35	0.65
NLR			
Continuous	1.09	0.99–1.19	0.07
Monocytes (10E9/L)			
Continuous	1.39	0.22–8.72	0.73
Platelets (10E9/L)			
Continuous	0.997	0.99–1.003	0.32
LMR			
Continuous	0.83	0.57–1.22	0.34
PLR			
Continuous	0.999	0.996–1.003	0.68
Alb (g/L)			
Continuous	0.92	0.84–1.01	0.09
CHO (mmol/L)			
Continuous	0.21	0.04–1.21	0.08
TG (mmol/L)			
Continuous	1.57	0.74–3.31	0.24
HDL^a^ (mmol/L)			
Continuous	0.11	0.02–0.82	0.03
>0.875 versus ≤ 0.875	0.15	0.04–0.54	0.004
LDL (mmol/L)			
Continuous	1.84	1.11–3.05	0.07
ApoA1^a^ (g/L)			
Continuous	0.03	0.002–0.40	0.008
>0.865 versus ≤ 0.865	0.14	0.04–0.50	0.003
ApoB (g/L)			
Continuous	0.39	0.02–7.31	0.53

HR, hazard ratio; CEA, carcinoembryonic antigen; CRP, C-reactive protein; LDH, lactate dehydrogenase; ALB, albumin; NLR, neutrophil-to-lymphocyte ratio; PLR, platelet-to-lymphocyte ratio; LMR, lymphocyte-to-monocyte ratio; CHO, cholesterol; TG, triglyceride; HDL, high-density lipoprotein; LDL, low-density lipoprotein; ApoA1, apolipoprotein A1; ApoB, apolipoprotein B.

a Optimal cutoff points were estimated by receiver operating characteristic (ROC) curve analysis.

**Table 3:** Multivariate survival analysis after variable selection for progression-free survival.

Clinicopathologic Parameters^#^	HR	95% CI	p-Value	HR	95% CI	p-Value
HDL^a^ (mmol/L)						
>0.875 versus ≤0.875	0.36	0.04–3.31	0.37	0.13	0.01–1.44	0.10
ApoA1 (g/L)						
>0.865 versus ≤0.865	0.28	0.03–2.67	0.27	0.56	0.06–5.26	0.61
Frequency of CD4+ T cell^a^ (%)						
>39.5 versus ≤39.5	4.83	1.28–18.27	0.02			
Ratio of CD4+/CD8+^a^ (%)						
>1.64 versus ≤1.64				9.23	2.04–41.69	0.004

HR, hazard ratio; HDL, high-density lipoprotein; ApoA1, apolipoprotein A1.

^#^Since frequency of CD4+ T cell was strongly correlated with ratio of CD4+/CD8+ with rho value of 0.73 (p < 0.001), these two parameters were separately included in the Cox model.

a Optimal cutoff points were estimated by receiver operating characteristic (ROC) curve analysis.

## Discussion

Anti-PD-1 immunotherapy is approved by the FDA for refractory dMMR CRC. In the present multicenter cohort study, the response rate for 41 patients with dMMR mCRC treated with anti-PD-1 inhibitors was analyzed, and the potential blood parameters were identified as predictive biomarkers for response. Although TMB has a potentially predictive value for anti-PD-1 therapy in MSI-H mCRC patients, the identification of peripheral blood biomarkers is crucial because the access of biomarkers from the blood is easier than that from tumor tissues. Considering the limited experience with anti-PD-1 therapy in patients with dMMR mCRC, the evidence of potential blood biomarkers for these patients was scarce. To the best of our knowledge, the present study with 41 patients with dMMR mCRC is the first multicenter study to show that the baseline level of the frequency of CD4+ T cell and the ratio of CD4+/CD8+ are independent potential biomarkers for ORR and survival in dMMR mCRC patients. Moreover, the present study indicated the potential prognostic value for NLR regarding OS.

Previously, pretreatment counts of peripheral blood cells or LDH have been investigated as potential biomarkers for clinical outcomes in patients with melanoma (10, 11, 19) and NSCLC (13, 14, 20) treated with ICIs. Even though the present study showed that NLR could potentially predict OS, it failed to indicate the predictive value of NLR for ORR and PFS in anti-PD-1 therapy, which indicated that dMMR mCRC might be distinct with melanoma or NSCLC. As far as we know, this study enrolled 41 dMMR mCRC patients firstly to show that pretreatment frequency of CD4+ T cell and the ratio of CD4+/CD8+ are independent potential biomarkers for both ORR and survival. Our analysis thus showed that a low ratio of CD4 T cell (≤39.5) was significantly associated with a better ORR and PFS/OS in dMMR patients with mCRC. The potential mechanism may be that CD4+ lymphocytes are anergised rather than being stimulated, which therefore correlate with a poor prognosis (21). Moreover, the domain type of the frequency of CD4+ T cell in the peripheral blood may be regulatory T cells, which have been recently reported to inhibit the antitumor activity of cytotoxic frequency of CD4+ T cell and then negatively affect the response and survival of patients undergoing anti-PD-1 immunotherapy (22). Moreover, the ratio of CD4+/CD8+ is a predictor for ORR and survival. This may be explained not only by the potential pro-tumor activity of the regulatory frequency of CD4+ T cell but also by the antitumor of the frequency of CD8+ T cell. More frequency of CD8+ T cells in the blood represents systematic antitumor immune features, and they could migrate to the tumor site, lymph nodes, and distal sites to enhance antitumor ability (17, 23, 24), which was consistent with the findings from a recent study (12) to investigate the peripheral blood cells to predict response to anti-PD-1 immunotherapy in melanoma. They found that the frequency of CD8+ T cells in the peripheral blood responders was reduced as compared with that in the blood of non-responders, which also indicated the crucial role in response to anti-PD-1 therapy. Moreover, the NICHE trial indicated that increased the frequency of CD8+ T cell counts in CRC might reflect an underlying immune activation (4).

Although our data revealed that HDL and ApoA1 were significantly associated with PFS and OS in a univariate analysis, the significance of HDL and ApoA1 was not maintained in a multivariate analysis. ApoA1, a prominent protein component in HDL, not only has antiapoptotic, anti-inflammatory, and antioxidant functions (25) but also alters tumor-associated macrophages (TAMs) from a pro-tumor M2 to an antitumor M1 phenotype (26) and modulates regulatory T cells (27). A recent study (18) also inferred that high ApoA1 correlated with higher TIL, which might be the reason for its potential positive impact on PFS.

The limitations of the present study include a relatively small sample size of patients and its retrospective nature. Another limitation lies in the absence of external validation of the associations detected in the present study, which need a further large-scale study to validate our findings. Since the present study has not performed the associations for other variables, especially TMB, which has been indicated to have a predictive value in dMMR cancers, future integrative analyses of circulating immune-based biomarkers with genomic and epigenetic biomarkers for clinical response or survival and prospective trials of MSI-H cancers are warranted to validate their predictive potential. In addition, the specific subtypes of peripheral leukocytes excluding CD4+ and CD8+ immune cells have not been analyzed, although these immune cells had different roles and prognoses in response to anti-PD-1 therapy. Thus, these findings require high content data-generating technologies to explore the potential mechanism for the circulating immune system and its correlation with the tumor immune microenvironment.

This is the first multicenter study to reveal that the frequency of CD4+ T cell and ratio of CD4+/CD8+ are biomarkers to predict the response to anti-PD-1 therapy and survival within a dMMR mCRC population. The finding indicates that patients with very low frequency of CD4+ T cell or low ratio of CD4+/CD8+ might respond well to PD-1 inhibitors, and this subset of patients might be further selected to receive first-line treatment with anti-PD-1 immunotherapy, which was consistent with the recent concept that anti-PD-1 immunotherapy is moved to first-line treatment for mCRC (28). These findings might provide a potential explanation for the variability in response to ant-PD-1 immunotherapy in numerous prospective clinical trials among dMMR mCRC patients and support the potential predictive role of the frequency of CD4+ T cell and ratio of CD4+/CD8+ in anti-PD-1 immunotherapy.

## Conclusion

In summary, this multicenter cohort indicated that the ratio of CD4+/CD8+ and the frequency of CD4+ T cell might be crucial independent biomarkers within dMMR mCRC to better identify patients for anti-PD-1 immunotherapy. If validated in prospective clinical trials, the ratio of CD4+/CD8+ and the frequency of CD4+ T cell might aid in guiding the treatment of PD-1 inhibitors among patients with dMMR mCRC.

## Data Availability Statement

The raw data supporting the conclusions of this article will be made available by the authors, without undue reservation.

## Ethics Statement

The studies involving human participants were reviewed and approved by the Medical Ethics Committee of the Sixth Affiliated Hospital of Sun Yat-sen University, Guangzhou, China (no. 2020ZSLYEC-216). The patients/participants provided their written informed consent to participate in this study. Written informed consent was obtained from the individual(s) for the publication of any potentially identifiable images or data included in this article.

## Author Contributions

Conception and design: PL, S-BY, and Y-KC. Financial support: PL, Y-KC, and S-BY. Provision of study materials or patients: PL and Y-KC. Collection and assembly of data: D-WC and PC. Data analysis and interpretation: S-BY, Y-KC, and D-WC. Manuscript writing: All authors. Final approval of manuscript: All authors.

## Funding

This study was supported by Key-Area Research and Development Program of Guangdong Province (2019B020229002), Science and Technology Planning Project of Guangzhou (No. 201902020009), the National Natural Science Foundation of China (Grant No. 81801111), and National Key Clinical Discipline, The National Key Research and Development Program of China (2017YFC1308800).

## Conflict of Interest

The authors declare that the research was conducted in the absence of any commercial or financial relationships that could be construed as a potential conflict of interest.

## Publisher’s Note

All claims expressed in this article are solely those of the authors and do not necessarily represent those of their affiliated organizations, or those of the publisher, the editors and the reviewers. Any product that may be evaluated in this article, or claim that may be made by its manufacturer, is not guaranteed or endorsed by the publisher.

## Abbreviations

dMMR, deficient mismatch repair; PD-1, programmed cell death protein 1; mCRC, metastatic colorectal cancer; PFS, progression-free survival; CEA, carcinoembryonic antigen; OS, overall survival; ORR, overall response rate; MSI-H, microsatellite instability-high; FDA, Food and Drug Administration; TMB, tumor mutational burden; NLR, neutrophil-to-lymphocyte ratio; ANC, absolute neutrophil count; CRP, C-reactive protein; LDH, lactate dehydrogenase; NSCLC, non-small cell lung cancer; ICIs, immune checkpoint inhibitors; PLR, platelet-to-lymphocyte ratio; LMR, lymphocyte-to-monocyte ratio; ALB, albumin; CHO, cholesterol; TG, triglyceride; LDL, low-density lipoprotein; HDL, high-density lipoprotein; ApoA1, apolipoprotein A1; ApoB, apolipoprotein B; HRs, hazard ratios; CR, complete response; PR, partial response; SD, stable disease; PD, progressive disease; TAMs, tumor-associated macrophages.
