# Supplementary material for: Association of Peripheral Blood Biomarkers With Response to Anti-PD-1 Immunotherapy for Patients With Deficient Mismatch Repair Metastatic Colorectal Cancer: A Multicenter Cohort Study
Source: Front Immunol. 2022 Feb 3;13:809971. doi: 10.3389/fimmu.2022.809971 (PMC8850282; doi:10.3389/fimmu.2022.809971)
Supplement: Supplementary file 1 [file Table_1.docx]

Supplementary Material

# Supplementary Tables

## Supplementary Tables

**Table S1. Clinical outcomes of the entire cohort**

| **Clinical outcomes** | **No. of patients (%)** |
| --- | --- |
| Complete response | 4 (10) |
| Partial response | 19 (46) |
| Stable disease | 10 (24) |
| Progressive disease | 8 (20) |
| Death | 8 (20) |

**Table S2. Overall survival and associations with clinicopathologic features using Cox regression**

| **Clinicopathologic parameters** | **HR** | **95%CI** | **P value** |
| --- | --- | --- | --- |
| Age (y) |  |  |  |
| Continuous | 1.03 | 0.99-1.08 | 0.17 |
| Gender |  |  |  |
| Female versus male | 1.03 | 0.25-4.14 | 0.97 |
| Location |  |  |  |
| Rectum versus colon | 0.55 | 0.13-2.33 | 0.42 |
| Grade |  |  |  |
| High versus moderate/ low | 0.038 | 0.0-16.09 | 0.29 |
| KRAS mutation |  |  |  |
| Yes versus no | 0.96 | 0.10-9.30 | 0.97 |
| BRAF mutation |  |  |  |
| Yes versus no | 0.04 | 0.00-10781.31 | 0.61 |
| Frequency of CD4+ T cell ^a^ (%) |  |  |  |
| Continuous | 1.09 | 1.02-1.18 | 0.019 |
| >39.5 versus ≤ 39.5 | 3.86 | 0.91-16.38 | 0.017 |
| Frequency of CD8+ T cell ^a^ (%) |  |  |  |
| Continuous | 0.91 | 0.83-1.001 | 0.054 |
| Ratio of CD4+/CD8+ ^a^ (%) |  |  |  |
| Continuous | 1.88 | 1.07-3.27 | 0.027 |
| >1.64 versus ≤ 1.64 | 6.82 | 1.37-33.99 | 0.019 |
| CEA (ng/mL) |  |  |  |
| Continuous | 1.002 | 0.999-1.005 | 0.17 |
| CRP (mg/L) |  |  |  |
| Continuous | 1.01 | 1.002-1.02 | 0.02 |
| > 2.21 versus ≤ 2.21 | 5.71 | 0.70-46.51 | 0.10 |
| LDH (U/L) |  |  |  |
| Continuous | 1.002 | 0.999-1.005 | 0.22 |
| Neutrophils ^a^ (10E9/L) |  |  |  |
| Continuous | 1.12 | 1.03-1.23 | 0.012 |
| >4.35 versus ≤ 4.35 | 9.08 | 1.11-74.21 | 0.05 |
| Lymphocytes (10E9/L) |  |  |  |
| Continuous | 0.56 | 0.15-2.12 | 0.39 |
| NLR |  |  |  |
| Continuous | 1.12 | 1.03-1.23 | 0.012 |
| >3.99 versus ≤ 3.99 | 13.28 | 1.62-108.70 | 0.016 |
| Monocytes (10E9/L) |  |  |  |
| Continuous | 1.38 | 0.15-13.06 | 0.78 |
| Platelets (10E9/L) |  |  |  |
| Continuous | 1.00 | 0.99-1.007 | 0.96 |
| LMR |  |  |  |
| Continuous | 0.82 | 0.52-1.29 | 0.39 |
| PLR |  |  |  |
| Continuous | 1.00 | 0.997-1.004 | 0.79 |
| ALb (g/L) |  |  |  |
| Continuous | 0.87 | 0.78-0.97 | 0.014 |
| >37.4 versus ≤ 37.4 | 0.29 | 0.07-1.16 | 0.08 |
| CHO (mmol/L) |  |  |  |
| Continuous | 0.32 | 0.06-1.64 | 0.17 |
| TG (mmol/L) |  |  |  |
| Continuous | 0.85 | 0.25-2.84 | 0.79 |
| HDL ^a^ (mmol/L) |  |  |  |
| Continuous | 0.06 | 0.005-0.76 | 0.03 |
| >0.875 versus ≤ 0.875 | 0.16 | 0.04-0.67 | 0.01 |
| LDL (mmol/L) |  |  |  |
| Continuous | 1.78 | 0.90-3.50 | 0.10 |
| ApoA1 ^a^ (g/L) |  |  |  |
| Continuous | 0.01 | 0.001-0.21 | 0.003 |
| >0.865 versus ≤ 0.865 | 0.097 | 0.02-0.40 | 0.001 |
| ApoB (g/L) |  |  |  |
| Continuous | 0.35 | 0.01-10.37 | 0.54 |

^a^ Optimal cut-off points were estimated by receiver operating characteristics (ROC) curve analysis.

HR= hazard ratio, CI= confidential interval, CEA=carcinoembryonic antigen, CRP=C-reactive protein, LDH=lactate dehydrogenase, ALB= albumin, NLR=neutrophil-to-lymphocyte ratio, PLR=platelet-to-lymphocyte ratio, LMR=lymphocyte-to-monocyte ratio, CHO=cholesterol, TG= triglyceride, HDL=high-density lipoprotein, LDL=low-density lipoprotein, ApoA1=apolipoprotein A1, and ApoB= apolipoprotein B.

**Table S3. The association between Frequency of CD4+ T cell or CD4+/CD8+ ratio with other potentially related factors**

|  | Frequency of CD4+ T cell, median | P value | Ratio of CD4+/CD8+, median | P value |
| --- | --- | --- | --- | --- |
| HDL (mmol/L) |  | 0.35 |  | 0.37 |
| >0.875 (n=31) | 36.0 |  | 1.30 |  |
| ≤ 0.875 (n=9) | 40.0 |  | 1.66 |  |
| ApoA1 (g/L) |  | 0.52 |  | 0.21 |
| >0.865 (n=34) | 40.0 |  | 1.25 |  |
| ≤ 0.865 (n=7) | 36.5 |  | 1.98 |  |
| NLR |  | 0.43 |  | 0.67 |
| >3.99 (n=17) | 36.0 |  | 1.16 |  |
| ≤ 3.99 (n=24) | 38.9 |  | 1.38 |  |

NLR=neutrophil-to-lymphocyte ratio, HDL=high-density lipoprotein, and ApoA1=apolipoprotein A1.

**Table S4. Multivariate survival analysis after variable selection for overall survival**

| **Clinicopathologic parameters**^#^ | **HR** | **95%CI** | **P value** | **HR** | **95%CI** | **P value** |
| --- | --- | --- | --- | --- | --- | --- |
| HDL ^a^ (mmol/L) |  |  |  |  |  |  |
| >0.875 versus ≤ 0.875 | 19769.9 | 0.00-2.15 E+137 | 0.95 | 738.67 | 0.00-5.81 E+98 | 0.95 |
| ApoA1 ^a^ (g/L) |  |  |  |  |  |  |
| >0.865 versus ≤ 0.865 | 0.00 | 0.00-1.14 E+128 | 0.94 | 0.00 | 0.00-1.27 E+92 | 0.94 |
| NLR ^a^ |  |  |  |  |  |  |
| >3.99 versus ≤ 3.99 | 32.48 | 1.86-568.57 | 0.017 | 19.31 | 1.60-233.20 | 0.02 |
| Frequency of CD4+ T cell ^a^ (%) |  |  |  |  |  |  |
| >39.5 versus ≤ 39.5 | 16.21 | 1.43-184.21 | 0.025 |  |  |  |
| Ratio of CD4+/CD8+ ^a^ (%) |  |  |  |  |  |  |
| >1.64 versus ≤ 1.64 |  |  |  | 15.22 | 2.00-115.80 | 0.009 |

^#^ Since Frequency of CD4+ T cell was strongly correlated with ratio of CD4+/CD8+ with rho value of 0.73 (p<0.001), these two parameters were separately included in the Cox model.

^a^ Optimal cut-off points were estimated by receiver operating characteristics (ROC) curve analysis.

HR= hazard ratio, CI= confidential interval, CEA=carcinoembryonic antigen, LDH=lactate dehydrogenase, LDL=low-density lipoprotein, ApoA1=apolipoprotein A1, and ApoB= apolipoprotein B.
